# Royal Infirmary, Glasgow

**Published:** 1831-03

**Authors:** 


### ROYAL INFIRMARY, GLASGOW.

*On Lithoplatomy.** By M. S. Buchanan, m.d., Member of the Faculty of Physicians and Surgeons, Glasgow, and one of the Surgeons to the Royal Infirmary.†

Case I. Mrs. Grant, aged fifty, of full habit of body and lax fibre; has been subject for many years to calculous complaints, but till lately would never submit to examination. On the 29th July last, was admitted under my care into the Royal Infirmary, and calculi of various sizes with ease detected lying at the neck of the bladder. In addition to the usual symptoms attendant on this painful disease, she had the most complete incontinence of urine I ever saw, the bladder having become much contracted, its coats thickened and constantly discharging white ropy mucus, from its internal surface. Extensive excoriation of nymphæ, labia, nates, and thighs, was the consequence; and considerable paralysis of inferior extremities, had of late rendered her a most pitiable object. Immediately on admission, I ordered her bowels to be well opened, anodyne injections to be administered, and the warm bath to be used; and on the 3d day of August, with consent of consultation, I dilated the urethra with Weiss' instrument, and extracted, with the aid of a small pair of forceps, three stones, two of them about the size of filberts, and the third, about the size of a walnut. She experienced little pain during the process of dilatation, which occupied about seven minutes. The incontinence of urine, and consequent excoriation of nates, &c. in a short time disappeared; and when she left the house, about three weeks after the operation, she could walk from one end of the ward to the other without assistance, her general health having at the same time greatly improved.

Case II. Robert Brock, a gentleman's servant, was admitted

* Λιϑος Lapis, and Πλατυνω, dilato. The operation of extraction of stone by dilatation.

† Glasgow Med. Journal.

under my care into the Infirmary, on the 11th August last. At that time, a stone could be felt in the urethra a little anterior to the bulb. It had advanced thus far about a fortnight previous to admission, and from its position now caused excruciating pain. He stated, that from his infancy, he had laboured under symptoms unequivocally indicating stone in the bladder, but, till lately, would not consent to be sounded. This operation was repeated with great care by several surgeons in this city, some months previous to admission, without any stone having been detected, and various means were also adopted the fortnight prior to the 11th instant, to get the stone dislodged from its situation in the urethra, by the introduction of catheters and bougies of the largest size, to the spot where it seemed sacculated; and also by the trial of various kinds of urethral forceps, assisted by the warm bath, &c. but all to no purpose. The gentlemen who met in consultation on his admission, were so convinced that no further trials of the above kind would succeed, that he was immediately placed on the operating table, secured as for lithotomy, a grooved staff introduced as far as the stone, and with one stroke of the knife, the urethra was incised to the proper extent, and the stone extracted. The operation was the affair of a moment, and was so free of pain, that the patient seemed astonished at having handed to him the cause of all his misery. The stone measured two inches and eight lines in its largest circumference, and one inch and ten lines in its smallest; it was convex on its one side, and flat on the opposite, with a small nodule in the centre of this last, indicating that it was the smaller segment merely of a larger stone, which must still be in the bladder. This was made more evident, not only from the very sharp edge of the flat surface above described, (which had very much the appearance of having been recently broken,) but also from the concentric layers of lithic acid, which were observed when sawn through its largest diameter by the lathe. On the 14th, the wound in the perineum was completely healed, and no symptoms of calculus remained. He has been frequently sounded since without any stone having been discovered, and, in consequence of feeling in every respect well, he was dismissed cured.

*Query* 1st. Is there any instance on record, in which a stone of such dimensions was passed thus far into the urethra from the bladder?

2d. In what manner shall we account for the section or fracture of this stone in the bladder, no instrument having at any time detected it, till its exit from this viscus?

3d. Why is the alleged remaining half unable to be discovered by the most careful sounding, as the first was, previously to its escape into the urethra?

From the consideration of these two cases, and of another upon which I lately operated with success for stone in the bladder in the Infirmary, a number of interesting reflections suggest themselves.

*Could an instrument not be contrived, which, adopting the principle of gradual and continued dilatation, might be applied, not only to the female, but also to the male urethra, and by this means supersede both lithotomy and lithotrity, in the great majority of cases of stone in the bladder? I feel quite convinced that it might.

The vagina, we all know, can be easily and effectually dilated by a most admirable contrivance, named by Weiss speculum vaginæ. This consists of three convex blades, each about six inches in length and four lines in breadth, connected by a handle and screw at right angles to the blades, and so constructed that, by revolving the handle on its axis, the three blades open regularly, and thus, without the smallest pain, they dilate the vagina so completely, that a full view of all the uterine region may be obtained. The instrument which I have thought of, and now got fabricated, by Mr. Norie, a respectable surgeons' instrument-maker in this city, is upon the same principle as the above, but of course different in size; and instead of being hollow, it has, for strength's sake, been made solid. By means of this speculum urethræ, or vesicæ, or rather lithoplatome, a full view of the bladder in the female is, with a properly directed light, enjoyed, and it combines all the advantages of the instruments now in use, with none of their disadvantages. But, granting that an instrument, such as my female lithoplatome, is so superior to those previously used for stone in the female bladder, will the same principle hold as to the male, either for the purpose of dislodging stones from the longer and smaller urethra of our sex, or from the more distant and inaccessible bladder? I think a review of the parts concerned, or an attentive observation of the unassisted efforts of nature herself, proves this point in a very satisfactory manner. Let us for a moment compare the two conduits; that of the female is about two, that of the male about eight inches in length. In diameter there is, upon an average, a difference of perhaps not more than a line or a line and a half; they are both lined with a similar mucous membrane, and surrounded by muscular fibres, destined to very similar functions. What then, I would ask, is there to prevent a lithoplatome, as long and as thick as the largest sized bougie, from being passed into the male bladder, and its three convex converging blades, as formerly alluded to, made to perform dilatation in a similar manner as in the female? We know very well how easily a straight sound can be passed into the male bladder, and also with what superior accuracy the size, form, situation, and even structure of calculi, are able by this instrument to be ascertained. Nothing, therefore, in the curvature of this canal, its structure, or situation, can for a moment stand in our way, reasoning from the analogy of parts. But again, what are we taught by un-

* We pass over some remarks which Dr. B. offers upon various methods that have been suggested by different surgeons, to extract calculi from the urethra.—Editor.

assisted nature in her efforts to effect the object intended; or, reasoning from pathology, what conclusion are we compelled to adopt? In the second case narrated, we find a stone of two inches eight lines in one circumference, and one inch ten lines in another, passing from the bladder of this boy as far as the spongy part of the urethra; and many other cases are on record of smaller calculi passing altogether by this canal: indeed, with the exception of that part of the canal at the prostate gland, and glans penis, I feel convinced that stones, of nearly as large a size, might spontaneously pass by the male as by the female urethra.

In stricture of the urethra, either spasmodic or organic, it has been observed that this canal is often very much dilated by the accumulation of urine behind the contraction; and if this last be timously incised, or overcome, how often is it the case that the parts regain their natural form, no incontinence of urine remaining? The inference is obvious.

What have been the diameters of the largest bougies or catheters, which have been used for stricture, or dilatation of the male urethra, I know not; but sure I am, that a half-inch tube can, with ease, be passed into the bladder in a full-grown man, whose prostate is sound; and when we observe, besides, that Sir A. Cooper's forceps, for the extraction of calculi from the bladder, by the urethra, are at least three lines in diameter, to what extent of dilatation must that urethra have been exposed, where a stone was by them grasped and extracted?

From all which, I think, I am justified in concluding, that in all cases of calculi in the male, the lithoplatome, such as I have described, can, with perfect ease and safety, be introduced into the bladder, and the urethra so far dilated, as to allow of the passage or extraction of stones of very large caliber.

As to the operation of incising the urethra, such as was practised by me in the case of the lad Brock, I have not the smallest doubt that such a procedure will for ever afterwards be superseded by the use of the lithoplatome; for, whatever scepticism may remain on the mind of the inveterate lithotomist, whether hypogastric, perineal or recto-vesical, or of the more ingenious, though, in my opinion, more dangerous, euthusiasts for lithotrity, still both must allow, that a calculus in the urethra, at whatever part it may lodge, will, with the greatest ease, by means of the lithoplatome, be expelled; and even the operations of these stone-breakers, in the same manner, be much facilitated, by the diminution of the sittings to which, in the great majority of instances, the unfortunate sufferers with urinary calculi are necessitated in their hands to submit.

But, if, notwithstanding all that has been advanced, it is denied that the male urethra is capable of dilatation in the manner suggested, another method remains to be discussed, of more importance by far than any to which I have hitherto adverted, and to which I am inclined more particularly to apply the term lithoplatomy.

The operation I allude to, is that by which the male urethra is converted into a female one, and thus the difficulty and danger of both lithotomy and lithotrity is at one step superseded by lithoplatomy.

For the purpose of effecting the first part of this operation, the patient must be secured as for lithotomy; a grooved staff is introduced into the bladder; the membranous part of the urethra near the prostate incised, to the necessary extent, to admit the lithoplatome; and then, having withdrawn the staff, the operation of dilatation of the remaining part of the urethra, prostate, and neck of the bladder, will be, with nearly the same ease, safety, and effect, accomplished, by means of my lithoplatome, as in the female.

That there are many objections to the above method of operating for stone, I must at once admit, and until I can give it a fair trial on the living subject, I shall not contrast it either with lithotomy or lithotrity: judging, however, from what I have already experienced in the female urethra, and on the dead subject, in the male, I am not the least afraid of the result. I shall only add further, that if the operation of lithoplatomy does come up to my expectations, and that of many judicious and talented surgeons here, to whom I have mentioned it, how much will suffering humanity be relieved, and the labours of the surgeon facilitated.

But lest it should be said that the operation of lithoplatomy which I have above described, and so strongly recommended, is nothing more than the apparatus major, the Marian method, that of Le Cat, Le Dran, or Pajola, let any one take a glance at these methods of operating, and the difference must at once strike him. It would extend this communication too much, to enter at large on this interesting subject, which, at some future opportunity, I intend to resume; but I must be allowed to say, that, in investigating the writings of a vast number of lithotomists, both ancient and modern, I can find nothing which, in the smallest degree, approaches to the operation I have above described. It is the principle, I once more repeat, of slow and continued dilatation, by the instrument which I have recommended, applied to the whole, or a small part, of the male urethra, for the extraction of calculi, which alone constitutes my improvement of this part of surgery.

In stricture of the urethra, could a very slender lithoplatome, introduced through the constriction, not be made to overcome this more safely and speedily than either the simple or armed bougie, or conical sound? If the instrument cannot be passed through the spasmodic, or organic contraction of this canal, could it not, by its introduction as far as the stricture, and kept steadily in its place, be made, by gentle dilatation, to overcome this disease? or at least allow of armed bougies, or sounds, being more safely made to act in accomplishing their object?*

* We deem it incumbent upon us to observe, that Mr. Keate has lately published a letter in the Medical Gazette, (December 25th, 1830,) in which he states that he conceived, and discussed with some of his friends, the identical plan now proposed by Dr. Buchanan, for converting, as he calls it, *the male*

